# Erratum: Huizeling, E.; et al. Age-Related Changes in Attentional Refocusing during Simulated Driving. *Brain Sci.* 2020, *10*, 530

**DOI:** 10.3390/brainsci11030286

**Published:** 2021-02-25

**Authors:** Eleanor Huizeling, Hongfang Wang, Carol Holland, Klaus Kessler

**Affiliations:** 1 Aston Neuroscience Institute, Aston University, Birmingham B4 7ET, UK; h.wang26@aston.ac.uk; 2 Aston Research Centre for Healthy Ageing, Aston University, Birmingham B4 7ET, UK; c.a.holland@lancaster.ac.uk

The authors would like to submit the following corrections to the published paper [1]. In the in-text description of Figure 5 on page 12, the “alpha” and “theta” labels have been mistakenly switched. The authors, therefore, wish to correct the description of Figure 5 in the first paragraph on page 12 from:

“Consistent with the sensor level analysis, Figure 5 displays higher frontal theta and alpha source power in the Single-Task compared to Sequential-Task conditions in the younger group (Panels A–D, left column), lower posterior theta power in the Single-Task compared to Immediate Switch condition in the older group (Panel C, middle column), and higher frontal alpha power in the Single-Task compared to Delayed Switch condition in the older group (Panel D, middle column).”

To the following: 

“Consistent with the sensor level analysis, Figure 5 displays higher frontal theta and alpha source power in the Single-Task compared to Sequential-Task conditions in the younger group (Panels A–D, left column), lower posterior alpha power in the Single-Task compared to Immediate (non-significant) and Delayed Switch conditions in the older group (Panel C-D, middle column), and higher frontal theta power in the Single-Task compared to Immediate (non-significant) and Delayed Switch condition in the older group (Panel A–B, middle column).”

This error is not present elsewhere in the manuscript, and does not affect the interpretation of results in the Discussion section, nor the scientific conclusions, where the correct labels have been used. The authors would like to apologize for any confusion and inconvenience caused to the readers by these changes. The manuscript will be updated on the article webpage, with a reference to this erratum.
