# Supplementary material for: Age-Related Changes in Attentional Refocusing during Simulated Driving
Source: Brain Sci. 2020 Aug 7;10(8):530. doi: 10.3390/brainsci10080530 (PMC7465308; doi:10.3390/brainsci10080530)
Supplement: Supplementary file 1 [file brainsci-10-00530-s001.pdf]

Supplementary material: Age-related changes in attentional refocusing during simulated driving

Eleanor Huizeling, Hongfang Wang, Carol Holland, & Klaus Kessler.

1. Methods Supplementary material

Table S1. Counterbalanced conditions

List 1	List 2	List 3
IS	IS	IS
IS	DS	DS
ST	IS	ST
IS	ST	DS
ST	ST	DS
DS	IS	DS
ST	ST	ST
IS	DS	IS
DS	ST	ST
DS	ST	ST
ST	DS	IS
IS	IS	ST

Order of trials by condition in each of the pseudorandomised counterbalanced lists. ST (Single Task), IS (Immediate Switch), DS (Delayed Switch).

2. RTs Supplementary material

2.1. Braking RTs and driving speed

One-way ANOVAs were conducted to compare age groups' braking RTs (in response to the vehicle ahead braking) and driving speeds when passing the road sign. Means of participants' median braking RTs are presented in Figure S1 and means of participants' median driving speeds are presented in Figure S2.

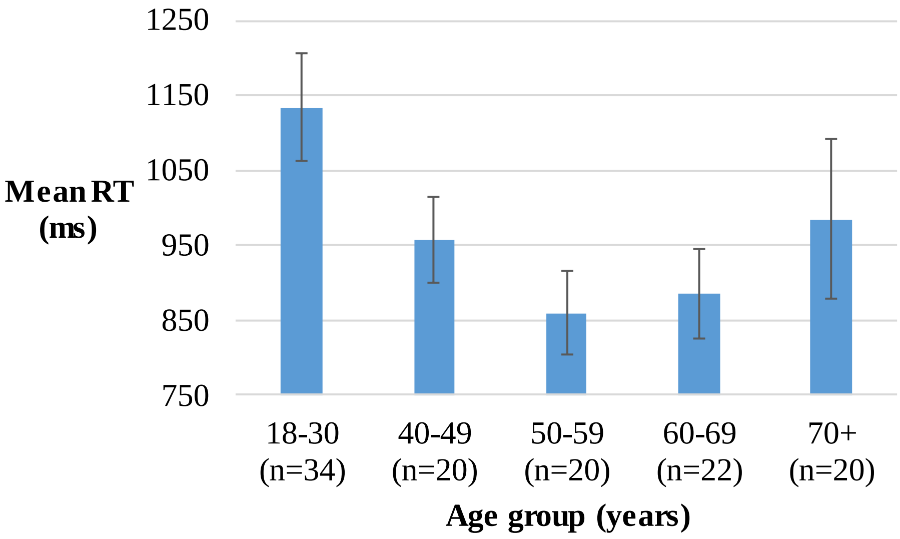

Figure S1. Group means of participants' median braking RTs. Vertical bars represent the SE.

A significant effect of age was found on braking RTs ($F(4, 115)=2.47$, $p=.049$). Braking RTs were faster in the 50-59 years group compared to the 18-30 years group ($p=.078$), however this did not reach significance. There were no other significant age group differences in braking RTs ($p>.10$).

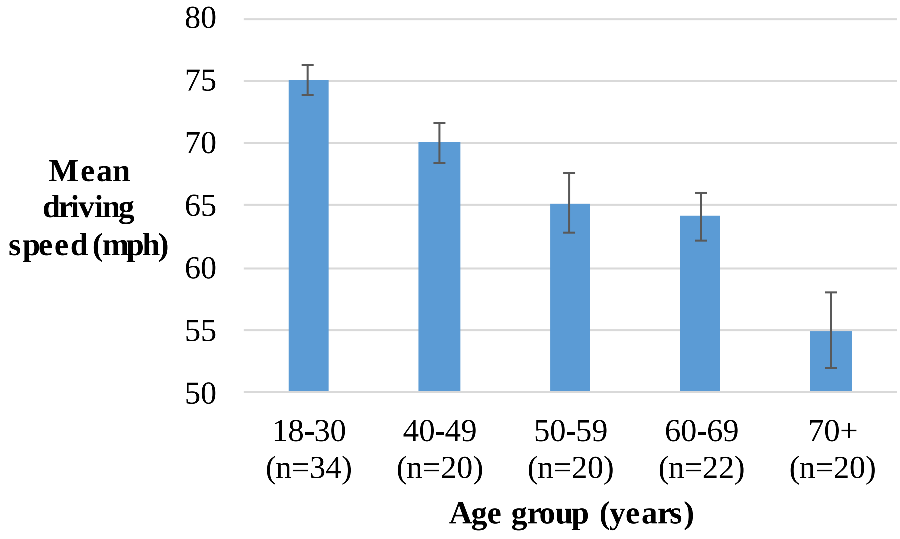

Figure S2. Group means of participants' median driving speeds when they passed the road sign. Vertical bars represent the SE.

A one-way ANOVA revealed age group differences in median driving speeds when participants passed the road sign ($F(4, 115)=15.44$, $p<.001$). The mean driving speed of the 18-30 years group was significantly higher than the 50-59 ($p=.004$), 60-69 ($p=.001$) and 70-91 years groups ($p<.001$).

3. EEG Supplementary material

3.1. TFRs

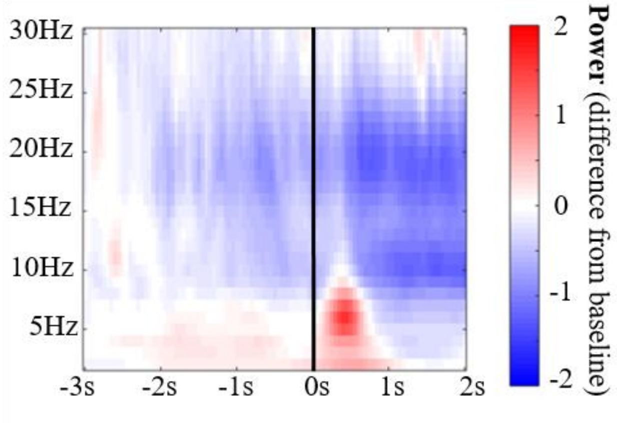

Figure S3. TFR in which time-frequency tiles for exploratory source analysis were selected, presenting power difference from a baseline period of −5.50 s – −3.50 s averaged across a group of 12 anterior electrodes (AF3, AF4, F1, F2, F3, F4, FC1, FC2, FC3, FC4, FC5, FC6) averaged across all conditions and all age groups. Black lines placed over TFRs signify the onset of the road sign at 0.00 s.

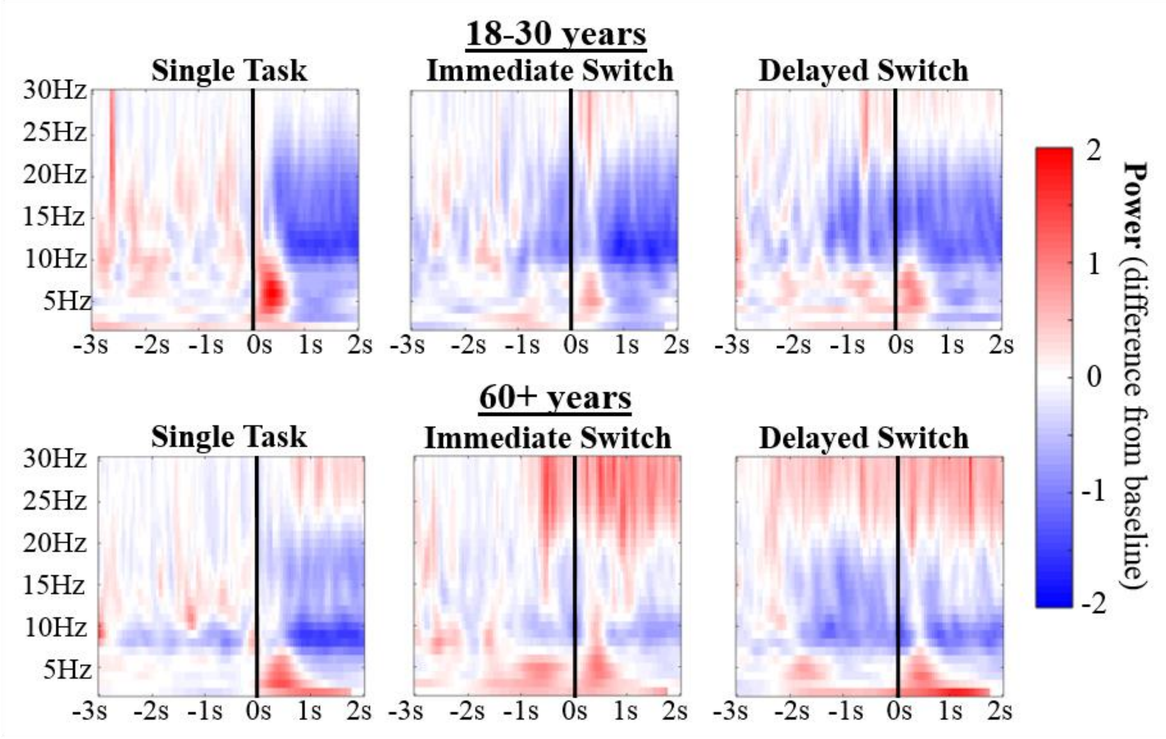

Figure S4. TFRs present power in relation to a baseline period of −5.50 s – −3.50 s averaged across a group of posterior electrodes (P7, P3, P4, P8, O1, O2, P5, P1, P2, P6, PO5, PO3, PO4, PO6, PO7, PO8). Black lines placed over TFRs signify the onset of the road sign at 0.00 s.

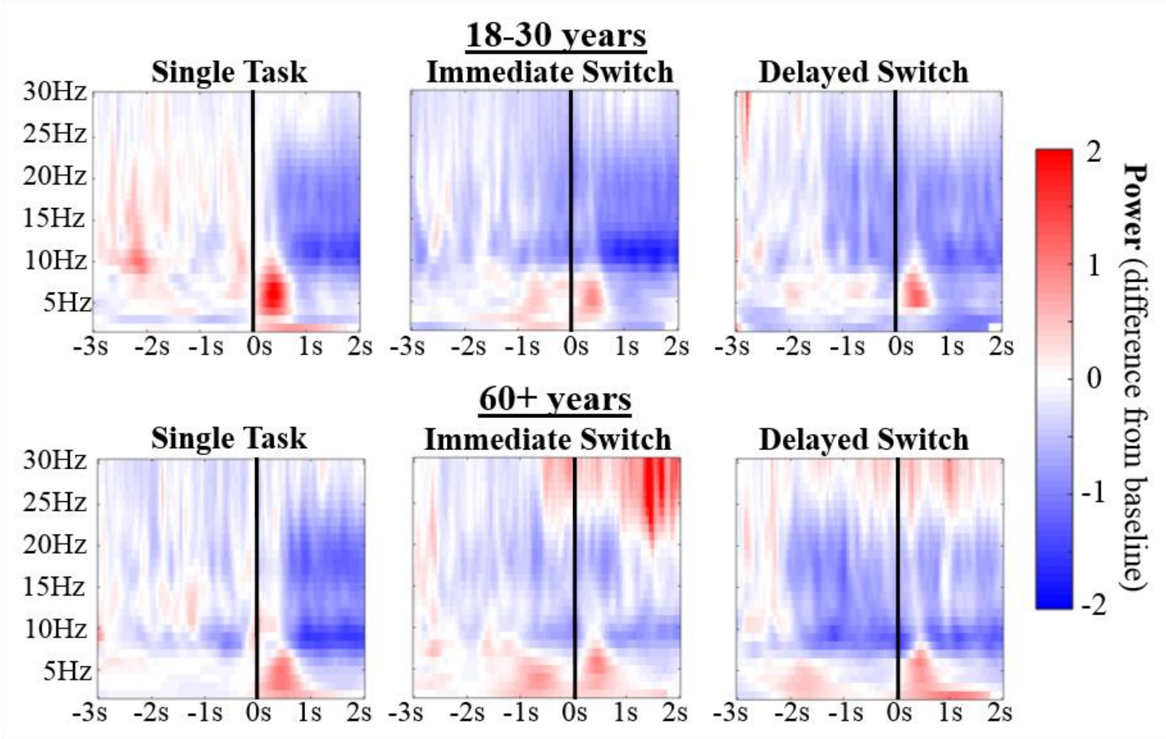

Figure S5. TFRs present power in relation to a baseline period of −5.50 s – −3.50 s in all electrodes that were included in the analysis. Black lines placed over TFRs signify the onset of the road sign at 0.00 s.

3.2. Indicator RT statistics in EEG group

To explore the behavioural effects in the subgroup of participants from which we recorded EEG (demographics in Table 1), a 3 × 2 (event condition × age group) ANOVA was conducted on participants' indicator RTs. Mean indicator RTs for each age group are presented in Figure 2B. The 60+ years group was significantly slower than the 18-30 years group ($F(1, 32)=38.75$, $p<.001$, $\eta^2_p=.55$). There was a significant main effect of event condition ($F(1.52, 48.68)=18.49$, $p<.001$, $\eta^2_p=.37$) on indicator RTs, but no age × event condition interaction ($p>.10$). Post hoc comparisons demonstrated that indicator RTs in the Single-Task condition were significantly faster than RTs in the two Sequential-Task conditions ($p<.001$). There was no significant difference between Immediate and Delayed Switch condition RTs ($p>.10$). Table 2 shows that, although Sequential-Task Costs are higher in the 60+ years group compared to the 18-30 years group, variability in Sequential-Task Costs is very high in this subsample, reflected in large SDs. It is likely that a combination of a small number of participants in the 60+ years group and high variability in Sequential-Task Costs across both age groups prevented group differences in Sequential-Task Costs from reaching statistical significance.
